# Root waving and skewing - unexpectedly in micro-*g*

**DOI:** 10.1186/1471-2229-12-231

**Published:** 2012-12-07

**Authors:** Stanley J Roux

**Affiliations:** 1 Section of Molecular Cell and Developmental Biology, University of Texas, Austin, TX, USA

**Keywords:** Arabidopsis, Auxin, Cytoskeleton, Extracellular ATP, International space station, Roots

## Abstract

Gravity has major effects on both the form and overall length of root growth. Numerous papers have documented these effects (over 300 publications in the last 5 years), the most well-studied being gravitropism, which is a growth re-orientation directed by gravity toward the earth’s center. Less studied effects of gravity are undulations due to the regular periodic change in the direction root tips grow, called waving, and the slanted angle of growth roots exhibit when they are growing along a nearly-vertical surface, called skewing. Although diverse studies have led to the conclusion that a gravity stimulus is needed for plant roots to show waving and skewing, the novel results just published by Paul et al. (2012) reveal that this conclusion is not correct. In studies carried out in microgravity on the International Space Station, the authors used a new imaging system to collect digital photographs of plants every six hours during 15 days of spaceflight. The imaging system allowed them to observe how roots grew when their orientation was directed not by gravity but by overhead LED lights, which roots grew away from because they are negatively phototropic. Surprisingly, the authors observed both skewing and waving in spaceflight plants, thus demonstrating that both growth phenomena were gravity independent. Touch responses and differential auxin transport would be common features of root waving and skewing at 1-*g* and micro-*g*, and the novel results of Paul et al. will focus the attention of cell and molecular biologists more on these features as they try to decipher the signaling pathways that regulate root skewing and waving.

## Commentary

In a recent paper published in *BMC Plant Biology*, Paul et al. [1] describe novel data that challenge a long-held hypothesis on how gravity affects patterns of root growth. When plants are grown on a solid surface, their roots show growth patterns of waving, which are undulations due to the regular periodic change in the direction root tips grow, and skewing, which is the slanted angle of growth roots exhibit when they are growing along a nearly-vertical surface. The generally accepted explanation for these patterns is that they result in large part from a combination of the touch stimulus arising from contact of the root tip with the surface and gravity, which increases the force of this contact. Using a specialized plant growth facility on the International Space Station, the Advanced Biological Research System (ABRS) with imaging hardware, Paul et al. collected digital photographs of plants every six hours during 15 days of spaceflight. The novel imaging system allowed the authors to observe the growth pattern of roots when their orientation was directed not by gravity but by overhead LED lights. Because the roots are negatively phototropic they grew away from the lights. In the micro-*g* environment of the ISS, plants grew more slowly than their 1-*g* controls grown under the same temperature and lighting on earth, but they still showed root waving and skewing. Their experimental design and controls made it unlikely that these growth patterns could be attributed to airflow, μ*g* vectors or other directional environmental factors.

Waving and skewing are largely surface-dependent phenomena, and do not appear in roots that grow embedded in agar [2]. Certainly, gravity-driven interactions of roots with surfaces influence waving and skewing, but these novel results of Paul et al. now make it clear that these growth patterns can also occur in micro-*g* when directional root growth that is driven by negative phototropism interacts with surfaces. Until these observations the only prior report of a root skewing pattern in micro-*g* was that of Millar et al. , who reported an inherent skew to the right in roots of Arabidopsis ecotype Landsberg grown in darkness [3]. In contrast the roots of Arabidopsis ecotype Columbia (Col-0) showed random growth in darkness [4]. To further document differences between ecotypes in their root growth patterns in micro-*g*, Paul et al. compared Col-0 and Wassilewskija (WS) ecotypes in their report, and found that both showed differences in their skewing and waving patterns in micro-*g*, just as they do on earth, but these differences were exaggerated in micro-*g*. Moreover, unlike the random pattern of root growth shown by Col-0 seedlings grown in darkness, the roots of Co-0 seedlings whose growth was oriented by negative phototropism showed both waving and skewing.

Unlike the earlier work of Millar et al. [3], Paul et al. captured a time-lapse record of the growth of two ecotypes in micro-*g* every six hours for 15 days, and were able to observe the root growth patterns that occurred during this period in response to a directional light gradient. Remarkably, the initial root growth of both ecotypes in both ground controls and spaceflight plants was straight away from the light for 5 days, and only then did the roots of the WS flight plants begin to show a strong 40^o^ skew to the right, whereas the WS ground controls began to skew only slightly; i.e., 10^o^ to the right. These results highlight the fact that skewing is dependent strongly on both stage of development and on the force of contact the root tip has to the surface, which is clearly less in micro-*g*.

Prior models proposed strong involvement of both gravity and touch as key stimuli for both the waving and skewing growth patterns of roots [2]. Clearly, the novel results of Paul et al. focus more attention on the role of touch stimuli for these phenomena. Both waving and skewing are differential growth phenomena, and current understanding would predict asymmetric auxin distribution would be a critical intermediate change needed for both. Mutants in the auxin transport facilitator PIN2 grow randomly, so do not show waving or skewing [5], and auxin transport inhibitors block the waving response [6].

This raises the question of what signaling steps or intermediate cellular changes link touch stimuli to changes in auxin distribution? Two answers favored in the literature are changes in the actin cytoskeleton and ethylene production. Touch stimuli alter cytoskeletal organization [2], which can strongly impact auxin transport [7]. Ethylene mediates both touch responses [8] and auxin transport [9]. Another, more speculative answer to how touch stimuli are linked to auxin transport changes arises out of several interrelated publications that have appeared recently. Weerasinghe et al. showed that when root tips experience touch stimuli they release ATP, and this release plays a role in redirecting root growth [10]. Extracellular ATP (eATP), which is now a recognized signaling agent in plants [11], induces rapid changes in [Ca^2+^_cyt_[12], and changes in Ca^2+^ transport can influence differential growth responses in roots [13]. These considerations predict that eATP could play a role in the control of auxin transport, and that applied nucleotides could alter skewing patterns, and both of these prediction have been confirmed experimentally (see Figures 1 and 5 in [14]).

## Conclusions

The data of Paul et al. provide novel and valuable documentation that the force of gravity is not needed for the waving and skewing patterns of root growth on solid surfaces, and that in micro-*g* these patterns differ between different ecotypes of Arabidopsis. They thereby focus more attention on the role of touch in these patterns, and especially on how the touch stimulus is linked to altered auxin transport, which is likely to be a key controller of waving and skewing in roots.

## Competing interests

The authors declare no competing interests

## Authors’ contributions

SJR conceived and wrote manuscript.
